# Non-immune Hydrops Fetalis and Hepatic Dysfunction in a Preterm Infant With Congenital Syphilis

**DOI:** 10.3389/fped.2019.00508

**Published:** 2019-12-11

**Authors:** Jessica Duby, Ari Bitnun, Vibhuti Shah, Patrick Shannon, Shiri Shinar, Hilary Whyte

**Affiliations:** ^1^Division of Neonatology, The Hospital for Sick Children, Toronto, ON, Canada; ^2^Department of Paediatrics, University of Toronto, Toronto, ON, Canada; ^3^Division of Infectious Diseases, The Hospital for Sick Children, Toronto, ON, Canada; ^4^Department of Paediatrics, Mount Sinai Hospital, Toronto, ON, Canada; ^5^Department of Pathology and Laboratory Medicine, Mount Sinai Hospital, Toronto, ON, Canada; ^6^Department of Laboratory Medicine and Pathology, University of Toronto, Toronto, ON, Canada; ^7^Department of Obstetrics and Gynaecology, Mount Sinai Hospital, Toronto, ON, Canada; ^8^Department of Obstetrics and Gynaecology, University of Toronto, Toronto, ON, Canada

**Keywords:** congenital syphilis, hydrops fetalis, hepatic dysfunction, pregnancy complications, infant-newborn

## Abstract

We report a case of a preterm infant with congenital syphilis who presented with non-immune hydrops fetalis. Hepatic dysfunction was present at birth and acutely worsened following antibiotic administration. Placental pathology demonstrated infiltration with numerous spirochetes. Although critically ill, the infant recovered with intravenous penicillin G and supportive care. This case demonstrates that congenital syphilis remains a contemporary disease demanding enhanced awareness from clinicians. Manifestations evident *in utero* or in the newborn can be severe and may result in fetal demise or neonatal death. Moreover, we hypothesize that the treatment resulted in a Jarisch-Herxheimer reaction as manifested by the hepatic deterioration. The incidence of congenital syphilis and its associated complications can be greatly reduced with strict adherence to universal prenatal testing and comprehensive follow-up.

## Introduction

Sir William Osler's adage—“he who knows syphilis knows medicine”—continues to ring true more than 100 years later given that infection with *Treponema pallidum* can mimic other diseases. Heightened clinical suspicion is needed, especially for congenital syphilis which can be fatal in its most severe form. We report a case of a preterm infant with congenital syphilis who presented with non-immune hydrops fetalis (NIHF) and hepatic dysfunction and was successfully managed with intravenous penicillin G and supportive care.

## Case Presentation

A male infant was born via Cesarean section at 31 weeks' gestation for a sinusoidal fetal heart rate tracing to a 28-year-old G5P2A2 woman with a history of gonorrhea in a previous pregnancy. She used cannabis recreationally but discontinued upon learning of her current pregnancy at 23 weeks' gestation. She received inadequate antenatal care with infectious diseases screening first being performed at the end of 30 weeks' gestation. In accordance with Ontario Public Health, syphilis serology screen using chemiluminescent microparticle immunoassay (CMIA) was completed, and the screen was positive^1^. The confirmatory *T. pallidum* particulate agglutination assay (TPPA) was also reactive and the rapid plasma reagin (RPR) showed a titer of 1:32. These results were obtained near the time of delivery precluding antenatal treatment. She also tested positive for *Trichomonas vaginalis*. Testing for hepatitis B & C, HIV, *N. gonorrheae* and *Chlamydia trachomatis* was negative.

Ultrasound at 31 week's gestation revealed normal fetal anatomy and hydrops fetalis. The middle cerebral artery peak systolic velocity (MCA-PSV) was elevated at 1.84 MoM. Fetal blood sampling confirmed anemia (55 g/L) as well as thrombocytopenia (7 × 10^9^/L), and *in-utero* packed red cell and platelet transfusions were completed. Maternal and neonatal blood types were both O Rh positive while Kleihauer-Betke test was negative for feto-maternal hemorrhage. Fetal echocardiography demonstrated a structurally normal heart with cardiomegaly. Additional antenatal bloodwork done as part of the workup for hydrops fetalis demonstrated non-reactive IgG and IgM for parvovirus B19, non-reactive IgG and IgM for toxoplasmosis, reactive IgG to CMV, indeterminate IgG to herpes simplex virus (HSV), and a protective rubella titer. Amniotic fluid was not tested prior to delivery.

At birth, Apgar scores were 1, 5 and 7 at 1, 5, and 10 min, respectively and the infant was intubated during resuscitation. The birthweight was 1,710 g (~50^th^%ile), length was 41 centimeters (~50^th^%ile) and head circumference was 30 centimeters (~75th%ile). Physical exam was significant for edema, ascites, hepatomegaly and petechiae. Transaminitis and hepatic dysfunction was present shortly after birth and worsened in the first 24–48 h (Table 1) along with elevated ferritin (peak > 40,000 μg/L). Peak derangements of INR (5.8) and aPTT (84 s) as well as the nadir of fibrinogen (<0.6 g/L) occurred at 48 h of life and was not associated with significant bleeding. The infant received one transfusion of fresh frozen plasma and was treated with a course of vitamin K. In addition, the infant required ventilator support for respiratory failure, vasopressors and hydrocortisone for hypotension, and multiple platelet transfusions for refractory thrombocytopenia. The infant also suffered from acute renal failure without urinary proteinuria, likely due to decreased renal blood flow as a result of the severe ascites. A peritoneal catheter was placed for drainage of recurrent ascites as well as renal replacement therapy.

**Table 1:** Liver function tests during the first week of life.

**Time since birth**	**ALT (U/L)**	**AST (U/L)**	**INR**	**Conjugated bilirubin (μmol/L)**	**Ammonia (μmol/L)**
2.5 h: antibiotics initiated					
3 h	622	3,830	–	64	–
6 h	1,260	–	2.7	–	–
12 h	1,680	>10,000	2.6	112	–
24 h	1,991	>10,000	5.4	188	82
48 h	943	–	5.8	201	151
72 h	365	–	2.5	261	127
96 h	161	828	2.8	210	45
1 week	88	92	1.7	88	17

The infant was investigated and treated empirically for congenital syphilis, receiving the first dose of antibiotics 2.5 h after birth. However, a broad differential diagnosis was considered given the unusual presentation. Due to concern for gestational alloimmune liver disease secondary to an elevated ferritin level, the infant received intravenous immunoglobulin. Subsequent MRI, however, did not show extrahepatic siderosis. Blood culture was negative as was blood PCR for CMV and HSV. Metabolic work-up and microarray were normal. Factor levels were not performed.

**Figure 1:**
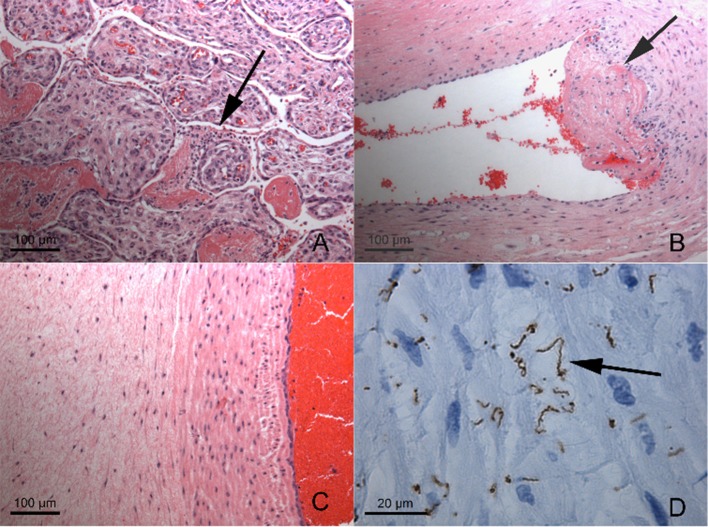
Placental Histopathology **(A)** Very scant intervillusitis (arrow) composed of granulocytes and lymphocytes between immature chorionic villi. **(B)** Mural thrombus formation (arrow) in large chorionic plate vessel, with scant mononuclear infiltrate (B and T lymphocytes with histiocytes) in vessel wall and deep thrombus. **(C)** Umbilical vein and adjacent cord, demonstrating lack of inflammation or necrosis. **(D)** Same field as **(C)**, immunohistochemical staining for *Treponema pallidum* demonstrating innumerable, large, often bent spirochetes (arrow). Magnifications as indicated by scale bars. There was no plasma cell deciduitis.

**Figure 2:**
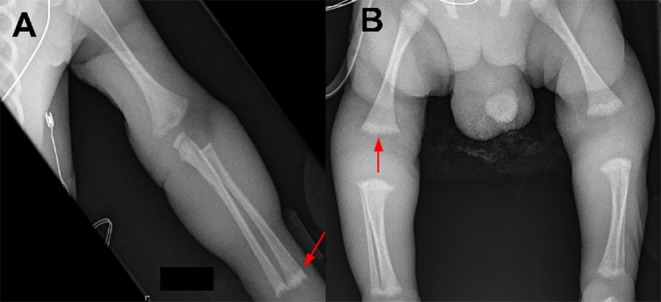
Long bone radiographic findings of a preterm infant with symptomatic congenital syphilis. **(A)** Sclerotic bands of the metaphyses (arrow). **(B)** Serrated epiphyseal plates (arrow).

Further testing confirmed the diagnosis of congenital syphilis. Placental pathology showed innumerable spirochetes but a lack of inflammation or necrosis (Figure 1). The infant's serum RPR titer was 1:16. Due to the infant's clinical instability, a lumbar puncture was only performed 1 week after birth and the cerebrospinal fluid (CSF) sample was bloody (RBC 179,000 × 10^6^/L; WBC 317 × 10^6^/L; glucose 3.2 mmol/L; protein 3.42 g/L). The CSF sample was negative by the venereal disease research laboratory (VDRL) test and indeterminate by the fluorescent treponemal antibody absorption (FTA-ABS) test. Brain MRI and ophthalmological exam were normal. Long bone x-rays revealed sclerotic bands along the metaphyses and serrated epiphyseal plates (Figure 2).

The infant improved with 14 days of intravenous penicillin G (50,000 units/kg/dose every 12 h for 7 days followed by 50,000 units/kg/dose every 8 h for 7 days) and supportive care (1). His liver dysfunction and kidney injury resolved, and he was discharged home at term corrected gestational age.

## Discussion

Syphilis is an ongoing global health concern. While the burden is highest in Africa (2), the incidence of syphilis has been steadily rising in Europe and North America over the past decade (3–5). Between 2010 and 2015, there was an 85% increase in the rate of syphilis in Canada although the incidence of congenital syphilis remained stable (1.54 cases/100,000 livebirths in 2015) (3). In the United States, on the other hand, the rise of syphilis translated to 918 infants born with congenital syphilis in 2017, the highest number of recorded cases in the country in 20 years (4).

Syphilis screening of pregnant women in the first trimester with treatment of those who test positive remains the standard of care to prevent congenital syphilis (1, 6). While universal re-screening of pregnant women in the third trimester is not cost-effective (7), both Canada and the United States recommend repeat testing mid-gestation and at the time of delivery in areas with heterosexual outbreaks of syphilis (1, 6). However, solely implementing enhanced prenatal screening recommendations during an outbreak may not be sufficient to slow the rate of congenital syphilis. Following a syphilis outbreak in 2009, Alberta increased the recommended number of prenatal syphilis tests, but after 2 years of the program, only 20.7% of pregnant women had completed all three tests (8). Risk factors for not completing the re-screening process were similar to known risk factors for contracting syphilis, including indigenous background and low socioeconomic status. Consequently, interventions that target those less likely to be screened are an important component of any public health response to rising rates of syphilis. An important message for all care providers is that no newborn should be discharged home prior to confirming the mother's syphilis screen results (1, 6).

Intramuscular penicillin G benzathine is 98% effective in preventing *in-utero* transmission of syphilis (9). In the absence of treatment, transmission is inversely related to the duration of the mother's disease with near universal transmission occurring with primary or secondary syphilis, 40% with early latent disease and < 10% with late latent disease (10). Transmission during the first and second trimesters is associated with a higher risk of early adverse outcomes including stillbirth, preterm delivery and symptomatic disease during the neonatal period as compared to third trimester infections. However, because transmission most commonly occurs later in pregnancy, most infants with congenital syphilis are asymptomatic at birth (10).

Congenital syphilis is an uncommon cause of NIHF in high-income countries and exemplifies Osler's observation of the infection's ability to imitate other diseases. Cardiovascular, chromosomal and hematologic abnormalities are much more common causes for NIHF (11). Congenital infections account for 5–7% of NIHF cases with parvovirus B19 and CMV being most common (11).

While NIHF is a known manifestation of congenital syphilis, only 1–6% of live born infants with congenital syphilis present with NIHF (12, 13). The presence of NIHF indicates an advanced stage of the infection. Transplacental transmission is hypothesized to cause hepatomegaly and placental abnormalities first, followed by amniotic fluid involvement and hematologic derangements prior to NIHF (14). NIHF from congenital syphilis is often fatal. However, *in-utero* resolution of NIHF has been described following maternal penicillin therapy with an intrauterine red blood cell transfusion (15). In all cases of NIHF, congenital syphilis should remain on the differential diagnosis.

The liver can be impacted in patients with congenital syphilis. Hepatomegaly is one of the most common manifestations secondary to extramedullary hematopoiesis (10, 12, 13). Cholestatic jaundice is present in 40–50% of symptomatic infants (12, 13). Both hepatomegaly and jaundice are typically transient but may take weeks to months to resolve. When congenital syphilis is fatal, autopsy may reveal treponemal infiltration of the liver tissue with or without fibrosis and inflammation that disrupt the lobar and biliary architecture (16, 17).

The severity and timeline of our patient's hepatic dysfunction uniquely highlights the impact that both congenital syphilis and its treatment may have on the liver. We hypothesize that the antimicrobial treatment given 2.5 h after birth potentiated our patient's hepatic damage (Table 1). Previous case series on congenital syphilis have documented a similar, transient hepatic deterioration following penicillin administration without evidence of spirochetes on subsequent liver biopsies (18, 19). The mechanism underlying this phenomenon may be endotoxin release from the lysed treponemes. Known as the Jarisch-Herxheimer reaction, this inflammatory response often causes pyrexia within a few hours following antimicrobial therapy for spirochetal infections (20), although this manifestation was not present in our patient. Another possible etiology for our patient's hepatic dysfunction is direct treponemal infiltration of the liver, but this is less likely to be the sole explanation given that treponemes are known to clear within hours after penicillin administration (21).

Infants whose mothers had reactive syphilis serology in pregnancy require syphilis serologic testing at birth (22, 23). Infants with suspected congenital syphilis should have a CBC with differential, long-bone x-rays, and CSF analysis with syphilis serology to evaluate for neurosyphilis. Ophthalmologic and audiologic assessment can be done selectively. Liver function tests should be considered, especially in the presence of hepatomegaly. Evidence of hepatic dysfunction should be monitored and may require further investigation if persistent.

Ten to fourteen days of intravenous penicillin G is required if the mother was inadequately treated, the infant's RPR titer is fourfold higher than the mother's titer regardless of maternal treatment, the infant has manifestations of congenital syphilis or treponemes are detected on skin lesions or bodily fluids (23). While placental pathology may be a valuable adjunct, it should not be the sole determinant to diagnosis congenital syphilis. There are no pathognomonic placental findings for congenital syphilis, and histopathological abnormalities may be present when the placenta acts as an effective barrier in preventing fetal transmission (24).

Patients with congenital syphilis require close follow-up. Syphilis serology should be repeated as a small percentage of children will need retreatment (22, 23). Infants with neurosyphilis require repeat lumbar puncture at 6 month intervals until normal with a low threshold for retreatment if the results remain abnormal (22, 23). Even with treatment, infants with congenital syphilis may develop long-term sequelae, including neurodevelopmental impairment (25).

## Conclusion

Our patient's unusual presentation of congenital syphilis reaffirms this infection's reputation as “the great imitator.” Congenital syphilis should be considered in the differential diagnosis of NIHF. Treatment for congenital syphilis may cause a transient worsening in hepatic function, especially if the liver is already compromised from the infection. Comprehensive prenatal care that detects and treats syphilis infection in pregnancy remains our greatest defense against congenital syphilis.

## Data Availability Statement

All datasets generated for this study are included in the article.

## Ethics Statement

Written informed consent was obtained from the minor(s)' legal guardian/next of kin for the publication of any potentially identifiable images or data included in this article.

## Author Contributions

SS provided obstetric care to the described patient's mother. PS completed the placental histopathology. JD, VS, AB, and HW provided medical care to the described patient. JD wrote the first draft of the manuscript and led revisions. All authors read, contributed edits, and approved the final version of the manuscript.

### Conflict of Interest

The authors declare that the research was conducted in the absence of any commercial or financial relationships that could be construed as a potential conflict of interest.

## Footnotes

^1^https://www.publichealthontario.ca/en/laboratory-services/test-information-index/syphilis-serology
